# A pilot study to compare swab versus fluid culture obtained from infected sites in the operating room

**DOI:** 10.1017/ice.2025.10353

**Published:** 2026-04

**Authors:** Monica Lou, Avvi Shabat, Priya Amin, Raymond Lopez, Kenneth Muldrew, Daniel Musher

**Affiliations:** 1 Baylor College of Medicine, Baylor College of Medicine, Houston, TX, USA; 2 Michael E. DeBakey Veterans Affairs Medical Center, Houston, TX, USA

## Abstract

Guidelines urge that infected fluid or tissue obtained during surgery be submitted for microbiologic study directly rather than via swab. A prospective study of operative specimens showed concordance in 64.7% of cases with better yield from abscess fluid, but swab cultures sometimes identified important pathogens missed by fluid culture.

## Introduction

Surgically obtained specimens from body sites that are thought to be clinically infected are regularly submitted from the operating room for Gram stain and culture. Gram stain results may be used to inform initial or empiric antibiotic therapy until final identification by culture is available, and results of culture can then be used for definitive antimicrobial therapy. In the absence of good microbiologic data, physicians often treat with overly broad or inappropriate antibiotic regimens.^1^ Microbiology laboratory usage guidelines^2,3^ state that fluid or tissue specimens are strongly preferred to swab specimens because swabs “hold extremely small volumes of the specimen, ... make it difficult to get bacteria or fungi away from the swab fibers and onto media, and the inoculum from the swab is often not uniform across several different agar plates.” Although this recommendation seems to be an excellent one, remarkably few studies have actually compared results of simultaneously obtained swab vs fluid or tissue cultures of operating room specimens.^4,5^ Accordingly, we designed a study to test the hypothesis that Gram stain and culture of infected fluid or tissue obtained in the operating room yield more valid data than Gram stain and culture of infected material submitted via swab. We specifically focused on abscesses, which we regarded as more problematic than, for example, specimens from infected joint fluid which has been extensively reported.

## Methods

In this “real-world” study conducted under IRB protocol at a Veterans Affairs Hospital, podiatric, vascular, orthopedic, and general surgeons were asked to submit for Gram stain and culture both a rayon swab in Amies agar gel transport medium (COPAN Diagnostics, Murrieta, CA) and infected fluid or tissue obtained at surgery from a site that was thought to be infected. Approximately 2 ml of fluid was collected by syringe and sent to the laboratory either in syringes or after being expelled into sterile containers. Tissue submitted in sterile containers was processed in sterile homogenizers. Specimens were studied by microscopic examination after Gram-staining and by culture on conventional media, including anaerobic culture if requested.

Results of Gram stain and culture of swab were compared to fluid and tissue as the “gold standard.” We evaluated concordance first between Gram stain and culture for each individual specimen and then compared results of Gram stain and culture from swab to those obtained from fluid or tissue specimens. Results of Gram stain and culture were regarded as concordant if the reading of predominant organism(s) by Gram stain identified the predominant organism(s) documented by culture. Results of culture of swab and fluid or tissue were regarded as concordant if same bacterial species were isolated from both or if both were negative. We discounted light growth of organisms unlikely to be implicated in infection such as coagulase-negative *Staphylococcus* species and diphtheroids.

## Results

A total of 71 pairs of specimens for which swab and fluid or tissue were studied by Gram stain and culture were collected from 68 patients who ranged in age from 28 to 92 years old. Sixty-three (93%) were male. Common comorbidities are shown in Figure S1. Samples were obtained from various anatomical sites (Table 1). Sixty-three of the 71 samples were from abscesses.

**Table 1.** Site of infection and type of sample

Anatomic site	Samples (*n* = 71)	Type of sample
Foot (*n* - %)	33 (46.5%)	30 abscess 2 synovial fluid 1 tissue
Leg (*n* - %)	10 (14.1%)	10 abscess
Abdomen (*n* - %)	6 (8.5%)	4 abscess 1 biliary fluid 1 hepatic cyst
Rectal/Gluteal (*n* - %)	6 (8.5%)	6 abscess
Groin (*n* - %)	5 (7.0%)	5 abscess
Axilla (*n* - %)	4 (5.6%)	4 abscess
Arm (*n* - %)	3 (4.2%)	2 abscess 1 synovial fluid
Sacrum (*n* - %)	1 (1.4%)	1 tissue
Oral (*n* - %)	1 (1.4%)	1 abscess
Chest (*n* - %)	1 (1.4%)	1 tissue
Intracranial (*n* - %)	1 (1.4%)	1 abscess

For 53 of 71 (74.6%) samples of fluid or tissue, Gram stain and culture showed the same predominant organism(s) (concordance), significantly greater than concordance in 39 of 71 swab samples (54.9%) (*P* = .014, by χ^2^ test) (Table 2). Discordance primarily reflected a negative Gram stain followed by culture growth, seen in 17 of 18 discordant fluid samples and 29 of 32 discordant swab samples. 15 paired samples were both discordant, 2 pairs showed discordance in fluid only, and 17 pairs showed discordance only in the swab.

**Table 2.** Results of Gram stain and culture growth by sample type

	Fluid/Tissue	Swab
Negative Gram stain and negative culture*	7 (9.9%)	10 (14.1%)
Positive Gram stain and positive culture	46 (64.8%)	29 (40.8%)
Negative Gram stain and positive culture	17 (23.9%)	28 (39.4%)
Positive Gram stain and negative culture	1 (1.4%)	4 (5.6%)

*7 samples showed no growth in culture for either sample.

In 11 of 17 (64.7%) cases in which culture results of swab and fluid or tissue differed, a bacterial pathogen was isolated from fluid or tissue but not from swab culture. In 4 (23.5%) cases, the swab culture yielded pathogenic bacteria that were not identified in fluid or tissue (*P* = .08 by McNemar’s test). In the remaining 2 cases, different pathogenic bacteria were isolated from cultures of fluid and swab (Figures S2 and S3).

## Discussion

Gram stain of infected material has the potential to provide a rational basis for initial selection of antibiotic therapy, a hallmark of antibiotic stewardship. The recently updated guideline,^3^ published jointly by the Infectious Diseases Society of America and the American Society for Microbiology reiterated,^2^ but without reference, that for microbiologic study “actual tissue, aspirates, and fluids are always preferred over swabs, especially from surgery,” and “Never use a swab ... from [a] surgical field if tissue can be submitted, [or] any body fluid,” Our study provides data that largely support this recommendation when fluid and tissue are able to be obtained, otherwise a swab is reasonable to obtain.

Our findings suggest that fluid samples may provide more accurate Gram stains than swabs. The sensitivity of Gram stain of a fluid or tissue sample in detecting a potential bacterial pathogen later grown in culture was 74.6%, compared to swab sensitivity of 55.2%. Bacterial pathogens were frequently identified by culture of fluid that were missed by culture of swab although in a few instances culture of swab specimens yielded important pathogens that were missed in culture of fluid. The lower sensitivity of the swab risks missing true infections. This suggests that multiple specimens submitted from an infected site are more likely than a single specimen—whether of fluid or of a swab—to yield positive results to guide therapy.

Collection and transport systems have been evaluated for at least half a century.^6^ Swabs of pus that use transport medium are clearly preferred to dry swabs,^7^ but comparisons of different swabs with different transport media have shown mixed results.^8–10^ Studies comparing tissue samples to swabs are similarly conflicting. Aggarwal et al found that tissue samples had higher sensitivity and specificity for prosthetic joint infection than swabs^4^ while Cherian et al noted higher inoculum in tissue but growth of different organisms in tissue and swab samples with unclear clinical significance.^5^ Mixed culture growth highlights that multiple culture samples might be best in every case, at risk of overloading clinical microbiology laboratories.

A strength of the present study is that it was a “real world” study, utilizing specimens as they were obtained in the operating room by operating room personnel. Had every specimen been obtained by a dedicated research team, there might have been a greater congruence in the results of different specimens, but such a result would not be generalizable. Limitations include reliance on a single swab collection system and the relatively small number of specimens obtained, although, even with this small number, significant differences could be noted.

In conclusion, submission of infected fluid or tissue yield more relevant Gram stain data than did submission of swab specimens, but our data suggest that submitting multiple types of specimens may provide more reliable culture results. Greater utilization of Gram stain results of liquid and tissue specimens may play an important role in antibiotic stewardship by enabling more directed selection of antibiotics.

## Supporting information

Lou et al. supplementary material Lou et al. supplementary material

## Supplementary material

The supplementary material for this article can be found at https://doi.org/10.1017/ice.2025.10353
