# Supplementary material for: A pilot study to compare swab versus fluid culture obtained from infected sites in the operating room
Source: Infect Control Hosp Epidemiol. 2026 Jan 23;47(4):421–3. doi: 10.1017/ice.2025.10353 (PMC13216795; doi:10.1017/ice.2025.10353)
Supplement: Lou et al. supplementary material [file S0899823X2510353Xsup001.docx]

|  | Patients (n = 68) |
| --- | --- |
| Age (median - range) | 65 (range 28-92) |
| Sex (male - %) | 63 (92.6 %) |
| Hypertension (n - %) | 45 (66.2%) |
| Diabetes (n - %) | 39 (57.4%) |
| Tobacco use (n - %) | 13 (19.1%) |
| Peripheral Artery Disease (n - %) | 11 (16.2%) |
| Stroke (n - %) | 6 (16.2%) |
| Coronary artery disease (n - %) | 22 (32.3%) |
| Congestive heart failure (n - %) | 13 (19.1%) |
| Cancer (n - %) | 9 (13.2%) |
| Chronic kidney disease/End-stage kidney disease (n - %) | 16 (23.5%) |
| Cirrhosis (n - %) | 4 (5.9%) |
| HIV (n - %) | 2 (2.9%) |

**Figure S1. Baseline demographic and clinical characteristics of patients included**

**Figure S2. Organisms isolated from paired samples.**

|  | **Fluid/tissue** | **Swab** |
| --- | --- | --- |
| **Gram-positive bacteria** |  |  |
| MRSA | 11 | 10 |
| MSSA | 15 | 15 |
| *Streptococcus agalactiae* | 7 | 7 |
| *Streptococcus anginosus* | 1 | 1 |
| *Streptococcus mitis* | 2 | 3 |
| Streptococcus viridans group | 1 | 0 |
| *Enterococcus faecalis* | 10 | 11 |
| *Enterococcus faecium* | 1 | 2 |
| *Enterococcus avium* | 2 | 1 |
| *Aerococcus urinae* | 1 | 0 |
| **Gram-negative bacteria** |  |  |
| *Serratia marcescens* | 1 | 1 |
| *Morganella morganii* | 2 | 6 |
| *Escherichia coli* | 10 | 9 |
| *Proteus mirabilis* | 5 | 3 |
| *Klebsiella pneumoniae* | 6 | 5 |
| *Klebsiella oxytoca* | 2 | 2 |
| *Klebsiella aerogenes* | 1 | 0 |
| *Pseudomonas aeruginosa* | 3 | 4 |
| *Citrobacter freundii* | 2 | 4 |
| *Citrobacter amalonaticus* | 1 | 1 |
| *Enterobacter cloacae* | 1 | 1 |
| **Anaerobes** |  |  |
| *Peptostreptococcus* sp. | 1 | 1 |
| *Prevotella* sp. | 3 | 0 |
| *Veillonella parvula* | 1 | 1 |
| *Bacteroides* sp. | 10 | 9 |
| *Parabacteroides* sp. | 2 | 2 |
| *Actinomyces* sp. | 2 | 2 |
| **Fungi** |  |  |
| *Candida glabrata* | 2 | 2 |
| *Candida albicans* | 1 | 1 |
| *Candida parapsilosis* | 1 | 1 |
| *Candida tropicalis* | 1 | 0 |
|  |  |  |
| **No growth** | 8 | 12 |
| **Monomicrobial** | 26 | 21 |
| **Polymicrobial** | 38 | 38 |

MRSA – Methicillin-resistant *Staphylococcus aureus*

MSSA – Methicillin-susceptible *Staphylococcus aureus*

sp. – species

**Figure S3. Description of discordant samples with different microbial isolates**

| **Type of infection sampled** | **Swab isolates** | **Fluid/Tissue isolates** | **Sample with additional isolate** |
| --- | --- | --- | --- |
| Leg abscess | *Enterococcus faecium*  *Morganella morganii* | *Enterococcus faecium* | Swab |
| Abdominal abscess | *Serratia marcescens* | *Serratia marcescens*  *Aerococcus urinae* | Fluid |
| Foot abscess | No growth | *Klebsiella pneumoniae* | Fluid |
| Foot abscess | MRSA  *Streptococcus agalactiae* | MRSA | Swab |
| Foot abscess | *Proteus mirabilis*  *Enterococcus faecalis*  *Providencia stuartii* | *Proteus mirabilis*  *Enterococcus faecalis* | Swab |
| Foot abscess | *Proteus mirabilis*  MSSA | *Proteus mirabilis*  MSSA  *Klebsiella pneumoniae* | Fluid |
| Brain abscess | No growth | *Streptococcus intermedius* | Fluid |
| Toe synovial fluid | No growth | *MSSA* | Fluid |
| Foot abscess | *Citrobacter sp*. *Enterococcus faecalis* | *Enterococcus avium* | Mixed |
| Groin abscess | No growth | *Prevotella* sp. | Fluid |
| Rectal abscess | *Proteus mirabilis*  *Escherichia coli*  *Bacteroides* sp.  MSSA | *Proteus mirabilis*  *Escherichia coli*  *Bacteroides* sp. | Swab |
| Leg abscess | No growth | *Prevotella bivia*  *Klebsiella aerogenes* | Fluid |
| Foot abscess | *Citrobacter* sp.  *Bacteroides* sp. | *Citrobacter* sp.  *Bacteroides* sp.  *Streptococcus gordonii* | Fluid |
| Foot abscess | *Citrobacter* sp. | *Bacteroides* sp. | Mixed |
| Leg abscess | MSSA | MSSA  *Escherichia coli* | Tissue |
| Chest wall abscess | *Escherichia coli* *Enterococcus faecalis* | *Escherichia coli* *Enterococcus faecalis*  *Proteus mirabilis* | Tissue |
| Sacral abscess | *Proteus mirabilis* | *Proteus mirabilis*  *MSSA* | Fluid |

MRSA – Methicillin-resistant *Staphylococcus aureus*

MSSA – Methicillin-susceptible *Staphylococcus aureus*

sp. – species
